# Urinary diversion after cystectomy: An Indian perspective

**DOI:** 10.4103/0970-1591.38611

**Published:** 2008

**Authors:** Deepak Jain, S. K. Raghunath, Samir Khanna, Prem Kumar, Sudhir Rawal

**Affiliations:** Department of Urology, Rajiv Gandhi Cancer Institute and Research Centre, Sector- V, Delhi - 110 085, India

**Keywords:** Radical cystectomy, urinary diversion, conduit, neobladder

## Abstract

Radical cystectomy remains the standard treatment for muscle-invasive carcinoma bladder. Various methods have been described for the urinary diversion. In the last 150 years urinary diversion has evolved from cutaneous ureterostomy to the orthotopic neobladder. Especially during the last 20 years, much advancement has been made. We hereby have reviewed the current approaches being used at different centers in India. We have also analyzed the evolution of diversion from conduit to the orthotopic substitution at our center.

## INTRODUCTION

Urinary diversion has a history of nearly 150 years.[1] In 1852, Simon performed the first ureteroproctostomy on a patient with exstrophy. The procedures have since become more refined and patient outcomes have improved.

In 1878, Smith performed ureterosigmoidostomy by directly anastomosing the ureters to the sigmoid colon. It was followed by creation of the rectal bladder by Gersuny in 1898. In the 1950s Bricker performed the urinary conduit formation with an isolated ileal loop. The first ileal neobladder was created by Camey in 1959 but orthotopic diversion gained much popularity only in the late 1980s.

Carcinoma urinary bladder has been the main cause requiring replacement of the bladder function. Radical cystectomy and pelvic lymph node dissection is the standard treatment for muscle-invasive organ-confined carcinoma of urinary bladder, a procedure initially popularized by Whitmore and Marshall.[2] Improved chemotherapy techniques have resulted in the increasing pool of operable patients.

Improved understanding of urodynamics has led to better configuration of reservoirs so that better storage is achieved without increasing the storage pressures.

Available options for replacement of bladder function are:

- Incontinent cutaneous diversions
- Continent cutaneous diversions
- Orthotopic (Ortho meaning correct, topic meaning of place) substitution

The Bricker ileal conduit has long been considered the gold standard for urinary diversion. Such a system drains continuously into a collecting device and allows free reflux into the collecting systems. In recent years, following the wide acceptance of clean intermittent catheterization, several procedures have been described for creation of a continent urinary reservoir.[3]

During the last decade there has been much interest in orthotopic neobladder reconstruction. This procedure, which requires a bowel segment, avoids an abdominal stoma and may offer an improved quality of life for patients undergoing radical cystectomy for bladder cancer.[4–7]

Over the past 15 years orthotopic reconstruction has evolved from “experimental surgery” to “standard of care at larger medical centers” to the “preferred method of urinary diversion” in both sexes, in developed countries.

The goals of orthotopic bladder replacement are to protect the upper urinary tracts and to allow the patient to void volitionally through the urethra in order to maintain a positive body and self-image.

## HISTORICAL REVIEW

The first successful urinary diversion following cystectomy was reported by Simon[1] in 1952. He diverted urine into the bowel in a patient of bladder exstrophy by joining the ureters to the rectum. This short-term success prompted the use of this technique in several other patients. Several complications were seen, especially relatively early postoperative deaths because of anastomotical incompetence and/or fecal reflux into the upper urinary tract.

Bringing the ureters to the skin i.e. cutaneous ureterostomy was tried next but it was found difficult to manage the continuous urine flow over the skin.[8]

Then efforts were made to divert the urine into the sigmoid colon.[9] Anal sphincter provided the excellent continence so it was the most commonly used type of diversion till 1950s.[10–12]

However, the risk of long-term complications with ureterosigmoidostomy (Hydronephrosis: 32%; pyelonephritis: 57%; metabolic derangements: 47%)[13] led to the search for other options.

The main risk found with ureterosigmoidostomy was septic complications. To avoid this, attempts were made to separate the feces from urine completely. Verhoogen (1908), Makkas (1910), Lengemann (1912) used the excluded ileocaecal segment as a reservoir and the appendix as an outlet valve.

In 1950, Bricker[14] used the isolated loop of ileum as a urinary conduit with a cutaneous stoma through which urine could be collected in a bag. Because of the easy construction and low rate of complications it soon became the gold standard for patients who underwent urinary diversion until the 1980s.

The main problem with the above-mentioned diversions was urinary incontinence, which severely affected the quality of life of patients. Gilchrist[15] and Merricks introduced in 1950 the concept of the continent pouch. It was simple because only intact anatomical structures were used: the caecum as reservoir and, instead of the appendix, the ileocaecal valve and the terminal anisoperistaltic segment of the ileum as antireflux mechanism. T Argentina by Gallo in 1946, by Santander in 1952 and by Mann and Bollmann in 1931 published the results of these techniques. The replication of good results was the main problem in using this continence mechanism and therefore it did not become very popular, however, the idea of the “continent skin stoma” still remains. Another technique to assure continence was first described in 1949 by Perl for a continent alimentary jejunostomy. The continence was achieved by invagination or intussusception of a segment of the small intestine. The principle was used by Ashken[16] and Mansson[17] among others with a caecal reservoir. The “hydraulic valve” with inversion of an ileal segment, described in 1974 by Benchekroun,[18] is based on the same principle: compression of the nipple valve by the surrounding fluid, which transmits the intraluminal pressure to the outlet valve. There were many disappointing results with continent suprapubic diversion, however, later, on retrospective analysis the main responsible factor for often urinary leak was not insufficient competence of the outlet valve, but high peristaltic properties causing high pressure peaks in intestinal reservoir. Ekman and Kock in 1964 first described the advantages of interrupting the tubular structure of intestine to make reservoir. Also Tasker and Giertz had clearly shown by then the superiority of Goodwin's cup-patch technique with four intestinal segments per cross-section area over the tubular reservoirs. In 1969, Kock[19] published the first results using Goodwin's cup-patch technique for reservoir and intussuscepted ileal nipple for continence to make an ileal continent fecal reservoir in patients after total proctocolectomy. Results of the same technique were also reported by Leisinger in 1976.[20]

Several investigators reported encouraging initial results with colonic reservoirs in the mid-1980s by applying the concepts of a cutaneous catheterizable ileocaecal reservoir which was developed in 1950[21–23] and simultaneously Kock *et al.*,[24] developed a catheterizable ileal pouch.

It was Camey and LeDuc[25] who reintroduced the concept of the neobladder in 1979 and other investigators improved the technique by applying the experiences of the various continent urinary diversions used earlier.[26–33]

Studer *et al.* and Hautmann used ileum to make low-pressure bladder substitution. Studer, after detubularizing the ileum, cross-folded the segment thus making it more spherical. He used the nondetubularized isoperistaltic segment of the ileum to prevent reflux of urine into the upper urinary tract.[34]

In 1994, Hautmann after publishing results with more than 200 ileal neobladders Using W fashioned ileum went on to conclude that ileal neobladder is the treatment of choice for male patients after radical cystectomy for the treatment of invasive bladder cancer.[35]

## INDIAN PERSPECTIVE

Table 1 shows patterns of urinary diversion used at different centers in India at present. It is obvious that in this country, ileal conduit is the preferred type of diversion over neobladder except at our center and at SN Medical College, Agra. Though reasons are unclear this difference seems to be because of experience gained with the large number of cases at the largest private cancer center in the country.

**Table 1:** Types of urinary diversion and the proportional use after cystectomy in different centers of the country

Center	No. of cystectomies per years	IIeal conduit %	Neobladder %	Mainz II %
AIIMS, New Delhi	50	81	8	5
TMH, Mumbai	70	60	40	0
RGCI and RC, New Delhi	52	30	70	0
GCRI, Ahmedabad	52	70	10	20
SNMC, Agra	10	10	90	0
IMS BHU, Varanasi	36	70	20	10
SVGCH, Miraz	20	60	35	5

Ileal conduit is an ideal diversion for most of the patients as it is easy to make, time taken is less and is easily managed postoperatively. Preoperative counseling is the most important part. Almost equally important is marking stoma preoperatively.

We have the experience of making over 350 urinary diversions at our exclusive cancer center in North India during the last 10 years. Diversion at this center has evolved from ileal conduit to neobladder. From 1996 to May 2000 all patients had diversion in the form of conduit. Ileal conduit was done in all patients except the patients who received radical radiotherapy preoperatively. In our country as radiotherapy is given by cobalt in most of the centers (which is not as precise as newer techniques like IMRT/3DCRT/IGRT), it causes extensive changes in small bowel and sigmoid which leads to poor healing if used in diversion. Transverse colon does not come to the pelvis and therefore can be safely used for diversion in such cases. We have done 18 transverse colon conduits. There had been no urinary or fecal fistula.

Though orthotopic diversion is more acceptable and has better mental quality of life, it has its own problem. It is technically more demanding, takes more time and the patient has to stay longer in the hospital. We started with using ileocaecal segment for neobladder and switched over to ileal segment. After finishing 36 ileocaecal neobladders we stopped doing ileocaecal neobladder because of more complications and more patients requiring intermittent clean catheterization.

## COMPLICATIONS ASSOCIATED WITH ILEOCECAL NEOBLADDER[36]

Urinary tract infections2Orchitis1Urinary leak12Urinary leak requiring repair4Deep vein thrombosis1Metabolic1Septicemia1Death1Bladder Outlet Obstruction4Subacute Intestinal Obstruction2

Ileal neobladder made by folding both limbs vertically after detubularization was used in four patients. However, in one of our patients we encountered difficulty in putting neobladder to urethra even by using all means of increasing the mesenteric length. This led us to innovate the neourethra.

Taking the incision for detubularization towards the mesentery in 4-5 cm of ileum in the most dependent part makes neourethra. With this approach we have observed that if the part of the ileum, which is touching the symphysis pubis, is used to make neourethra it will definitely reach comfortably to anastomosis without using other means of lengthening the mesentery.

**Figure 1:**
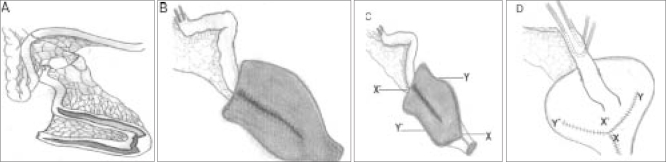
Pitcher pot ileal neobladder. (A) Excision of 55 cm ileal segment at least 25 cm proximal to ileocaecal junction. Distal 40 cm is opened along antimesenteric border except at apex of ‘U’ where it is opened towards mesenteric border. (B) Completion of posterior plate (C) Neourethral tube constructed. X′, proximal most end of posterior longitudinal suture line; X, proximal point of anterior suture line making neourethral tube; Y′, mid point of anterior wall of the distal detubularized segment; Y, Mid point of anterior wall of the proximal detubularized segment. (D) X′ and X are sutured by rotating X′ end of the detubularized segment to X point. Y0 to X0 and X0 to Y sutured after completion of uretero-intestinal anastomosis thus completing the neobladder construction. Ureteral stents are passed and brought out through the mesentery of the Studer's limb.

Another observation we have made is that by incorporating the distal cut end of the ileum into the neobladder capacity, though adds to it, is associated with prolonged urinary leak from neobladder and therefore we have stopped doing this as described in our original article. Figure 1 A, B, C, D show the neobladder we make at our center. With neourethra and double folding it gives the shape of Indian earthenware (Pitcher's Pot). Using this technique we have no patient who has required intermittent clean catheterization in 50 patients. This led us to conclude that the most important factor for satisfactory voiding following neobladder formation is tensionless anastomosis between neobladder and urethra.

However, there is apprehension that this neourethra may lead to high-pressure voiding and kinking of the tube can lead to retention of urine. We did Cystometrogram (CMG) in four patients and found that none of these was having high pressure during storage or during voiding. Two of our patients out of 50 were having overflow incontinence. On cystoscopy we found coapting mucosal fold causing obstruction in voiding. After resecting the mucosa patients voided well with insignificant residual urine. One of these patients required second time resection.

Uroflowmetry findings were as follows:

Mean Q max 19.06 ml/sec (range 7.5-43.8 ml/sec)

Mean Q ave 7.67 ml/sec (range 1.9-18.7 ml/sec)

Mean post-void residual urine 25.4 ml (range 10-62 ml)

Mean voided volume 299.12 ml (range 144-822 ml)

**Figure 2:**
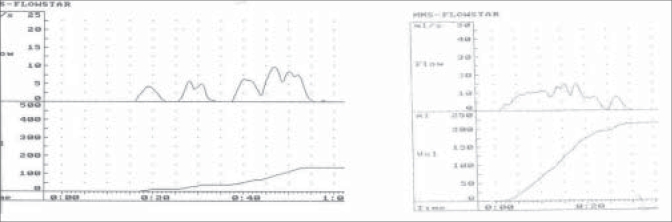
Uroflow showing various voiding patterns.

There were two types of voiding patterns, either continuous voiding pattern with bell-shaped curve or abdominal straining pattern [Figure 2].

In high-volume centers the trend is towards orthotopic diversion. We started doing orthotopic reconstruction since 1999 and there is an increase in the number of orthotopic diversions consistently [Table 2].

**Table 2:** Change in type of urinary diversion at Rajiv Gandhi Cancer Institute and Research Center from 1996-2006. These figures are of diversion used exclusively after cystectomy done for carcinoma bladder. The rest of the diversions were done for cystectomy done for other than bladder cancer (number not shown in table)

	1996	1997	1998	1999	2000	2001	2002	2003	2004	2005	2006	Total
Radical cystectomy	03	13	18	18	18	20	26	48	32	29	43	268
IIeal conduit	04	12	18	14	14	12	11	27	17	11	11	151
Colonic conduit	×	01	02	01	01	01	02	3	2	2	3	18
Sigma rectum pouch	×	01	01	×	×	×	×	×	×	0	1	03
Neobladder	×	×	×	03	04	08	15	22	13	18	35	118
Cut. ureterostomy	×	01	×	×	01	01	01	01	02	1	0	08
Ureterostomy	×	01	×	×	04	×	04	02	01	1	0	13
Ant. extentration	01	02	03	×	01	02	03	05	02	4	7	30

## CONCLUSION

There is no ideal urinary diversion till now. Every diversion has its pros and cons. Comparing urinary diversion in their physical and mental component score, patients with ileal conduit have a statistically and clinically significant decreased mental quality of life compared with age and sex-matched population. The type of urinary diversion after radical cystectomy significantly impacts the patient's quality of life.[29] Though neobladder gives a good body image and sense of voiding preservation, it is associated with nocturnal incontinence in up to 40% and with intermittent clean catheterization required to empty the bladder in up to 15%. This diversion cannot be used in 100% of patients as its prerequisite is cancer-free cut urethral margin on frozen section. Therefore, we not only require refinement of the existing technique but also require further innovations so that the patient can have the experience of close to normal voiding.
